# A case of colon cancer implanted on endoscopic resection ulcer certified by cancer genomic testing

**DOI:** 10.1007/s12328-024-02037-3

**Published:** 2024-09-26

**Authors:** Yuji Urabe, Hidenori Tanaka, Hikaru Nakahara, Fumiaki Tanino, Ken Yamashita, Shintaro Akabane, Akira Ishikawa, Manabu Shimomura, Hideki Ohdan, Shiro Oka

**Affiliations:** 1 Department of Gastroenterology, Hiroshima University Hospital, Hiroshima, Japan; 2 Department of Gastrointestinal Endoscopy and Medicine, Hiroshima University Hospital, Hiroshima, Japan; 3 Department of Clinical and Molecular Genetics, Hiroshima University Hospital, 1-2-3, Kasumi, Hiroshima, Minamiku 734-8551 Japan; 4 Department of Gastroenterological and Transplant Surgery, Graduate School of Biomedical and Health Sciences, Hiroshima University, Hiroshima, Japan; 5 Department of Molecular Pathology, Graduate School of Biomedical and Health Sciences, Hiroshima University, Hiroshima, Japan

**Keywords:** Colorectal cancer, Implantation recurrence, Cancer genomic testing

## Abstract

A 90 year-old man underwent endoscopic mucosal resection for lesions in the descending and sigmoid colons as well as endoscopic submucosal dissection (ESD) for a lesion in the rectal peritoneal reflection (Ra) 1 month before undergoing laparoscopic resection and D3 dissection for advanced cancer in the descending colon. One year later, he underwent a surveillance colonoscopy, and advanced colorectal cancer was detected on the ESD scar. The history suggested that this newly detected recurrent colorectal neoplasm on the ESD scar may have originated from cancer cells derived from the descending colon cancer that were implanted in the ESD ulcer, thereby initiating a new colorectal neoplasm. Cancer genomic testing further indicated that three of the four pathogenic variants detected in the recurrent colorectal neoplasm were consistent with pathogenic variants of descending colon cancer. This finding strongly supports our contention that cancer cells derived from the descending colon cancer were implanted in the post-ESD ulcer of the rectal Ra and proliferated, forming the recurrent colorectal neoplasm. This case report highlights the potential for tumor cell implantation on endoscopic resection ulcers and the utility of cancer genomic testing in validating this phenomenon.

**Supplementary Information:**

The online version contains supplementary material available at 10.1007/s12328-024-02037-3.

## Introduction

Transplantation of exfoliated cancer cells is recognized as one of the mechanisms underlying tumor recurrence at the suture site after colorectal cancer surgery [1, 2]. It is postulated that cancer cells floating in the intestinal tract settle and proliferate in the surgically exposed tissue, resulting in the development of recurrent lesions [3]. Endoscopic submucosal dissection (ESD) is one of the most widely used minimally invasive procedures for treating colorectal neoplasms [4]. In patients with advanced colorectal cancer, treatment of concurrent colorectal tumors often precedes surgery for advanced colorectal cancer. In this report, we present a case in which cancer cells originating from descending colon cancer had implanted within an ulcer following ESD of a colorectal neoplasm, and cancer recurrence was clarified via cancer genome analysis.

## Case report

**Fig. 1:**
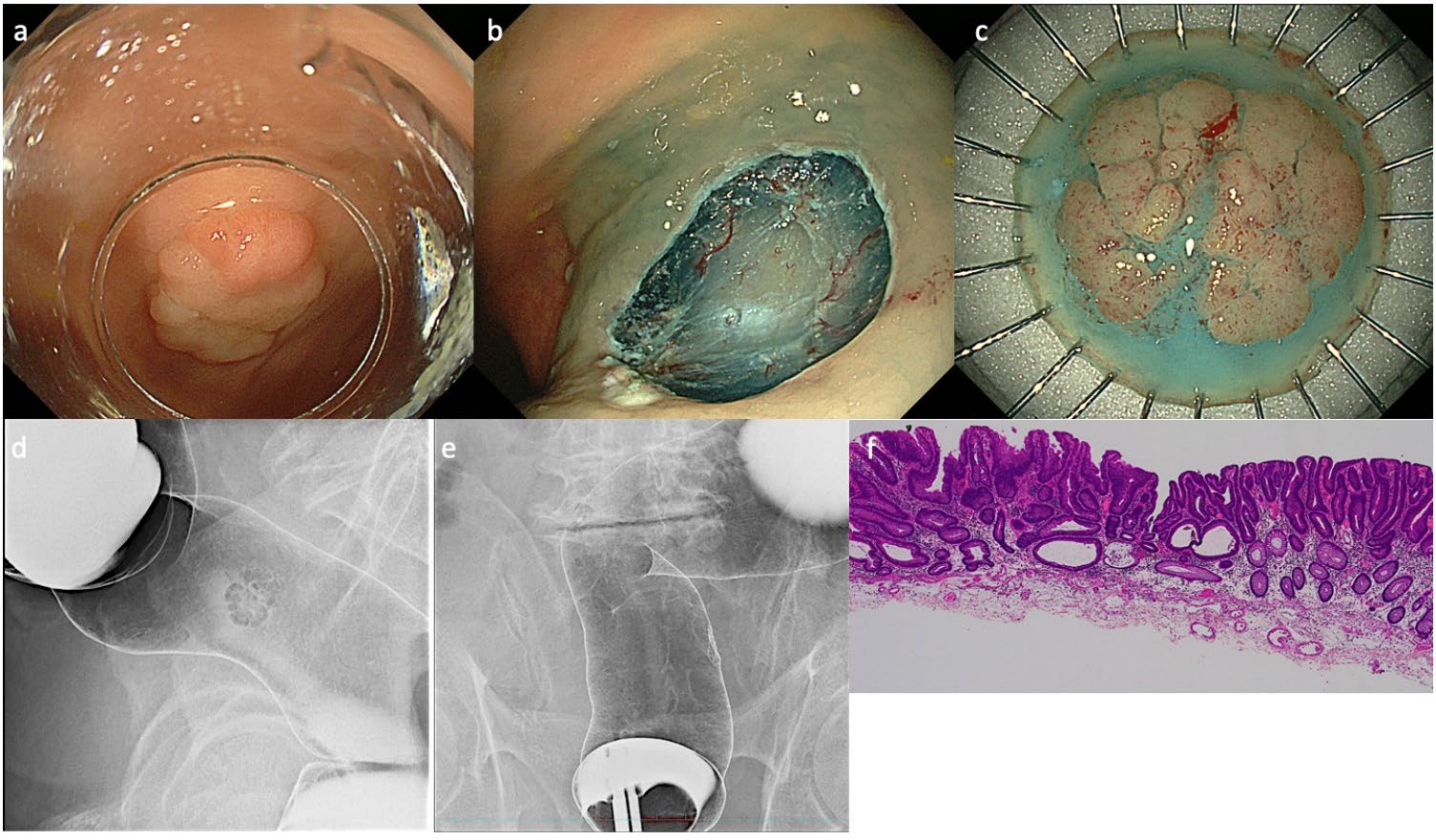
Clinicopathological findings of lesion A. **a** Lesion A was located in the rectum above the peritoneal reflection. This lesion was observed as a flat, elevated-type lesion measuring 20 mm in size on white-light imaging. **b** Ulcer immediately after endoscopic submucosal dissection. **c** Resected specimen. **d** Frontal view of lesion A on barium colonography. **e** Lateral view of lesion A on barium colonography. **f** Tumor tissue observation reveals tubular adenoma with severe atypia, high grade

**Fig. 2:**
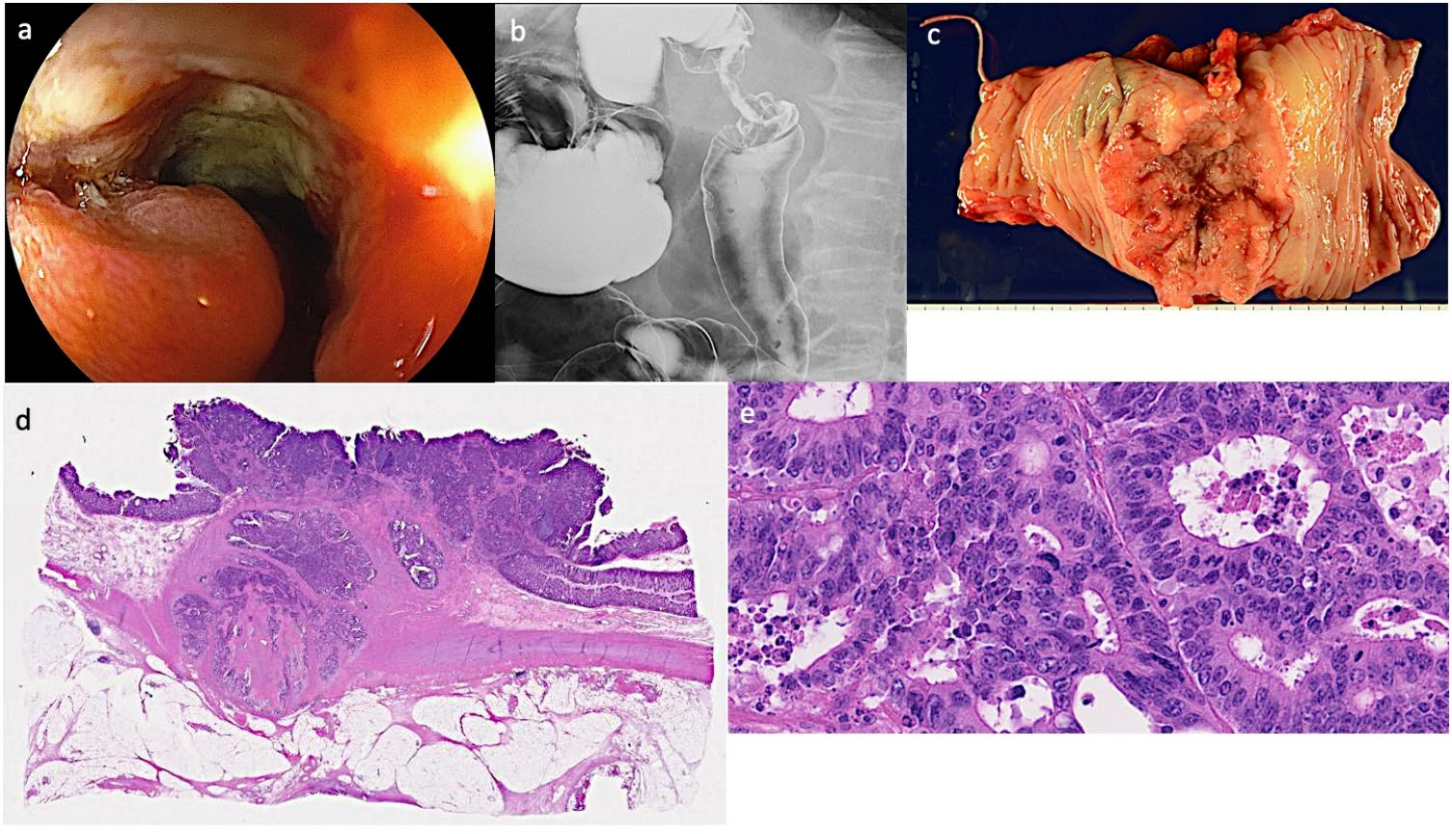
Clinicopathological findings of lesion B. **a** Lesion B was located in the descending colon. This lesion was 30 mm in size in type 2 advanced cancer. **b** Barium colonography of lesion B. The lesion shows the apple core sign. **c** Macroscopic view of the surgically resected specimen of lesion B. **d** Tumor tissue observation reveals fusion and infiltration of small and large atypical glandular ducts from the superficial layers to the sub-serosal tissues. Venous invasion, lymphatic invasion, and intramural nerve invasion are observed. Tumor clusters are mild. Tumor metastasis is also observed in the lymph nodes. **e** High-magnification view of the pathology for lesion B. Atypical gland ducts corresponding to moderately differentiated adenocarcinoma are seen

**Fig. 3:**
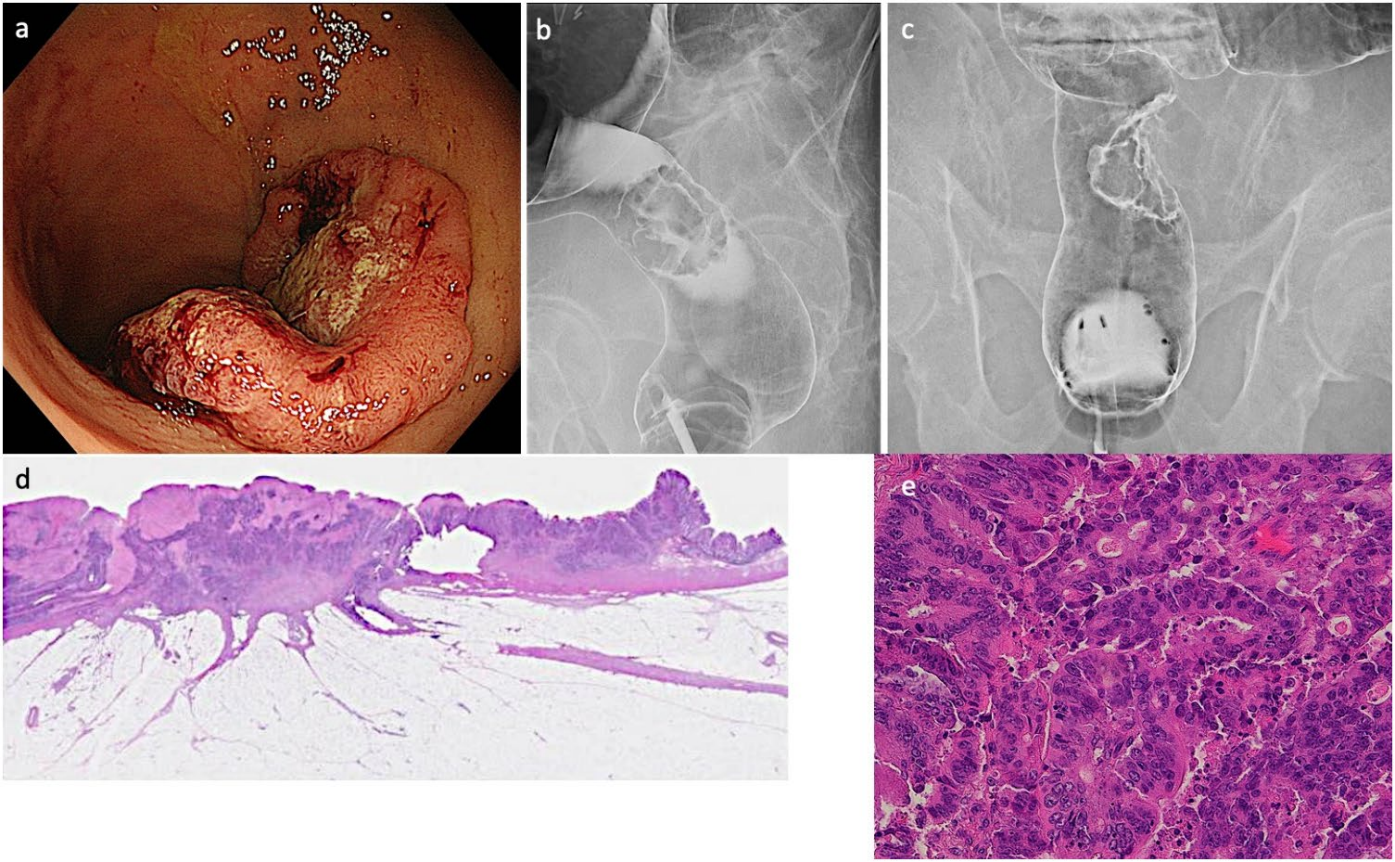
Clinicopathological findings of lesion C. **a** One year later, a 30-mm type 2 advanced cancer was observed on the ESD scar of lesion A. **b** Frontal view of lesion C on barium colonography. **c** Barium colonography showing a shadow defect on the lateral image of the right lateral wall of the Ra. **d** Sections show tumor tissue with moderately large or small irregular ducts fused in some areas and infiltrating from the mucosa to the adventitia. Most of the ductal adenocarcinomas are well-differentiated ductal adenocarcinomas with a poorly differentiated noncomplex component in the advanced areas. Venous invasion is also observed. Fibrosis is present in the deeper layers of tumor cells. **e** High-magnification view of the pathology for lesion C. Atypical gland ducts similar to those in lesion B are seen

We present the case of a 90 year-old man diagnosed with rectal cancer. In December 2018, he presented with back pain, and computed tomography (CT) findings revealed wall thickening at the splenic flexure. He was referred to our hospital for a colonoscopy, which showed a circumferential neoplastic lesion in the descending colon and multiple colorectal tumors. Colonoscopy revealed a 30 mm type 2 advanced cancer in the descending colon (**Lesion B**), a 10 mm 0-Is lesion in the descending colon, a 6 mm 0-Is lesion in the sigmoid colon, and a 20 mm 0-IIa lesion in the rectum above the peritoneal reflection (Ra) (**Lesion A**). Before surgery for advanced cancer of the descending colon, endoscopic mucosal resection (EMR) was performed on the 10 mm 0-Is lesion in the descending colon and 6 mm 0-Is lesion in the sigmoid colon, while ESD was performed on the 20 mm 0-IIa lesion on the Ra (**Lesion A**, Fig. 1). No clipping or other suture treatments were performed at the bottom of the ulcer. Pathological examinations of these three lesions revealed tubular adenomas with negative vertical and horizontal margins. One month after endoscopic treatment for colorectal neoplasms, he underwent laparoscopic resection of the descending colon and D3 dissection for advanced cancer. The pathological findings were as follows: tub2 > tub1, pT3(SS-A) INF b, Ly 1a, V1a, Budding grade (BD) 1, Pn1a, pN1b, pM0, and pStage IIIb (**Lesion B**, Fig. 2). One year after surgery for descending colon cancer, surveillance colonoscopy revealed type 2 advanced cancer in the rectum. Colonoscopy revealed a semi-peripheral ulcerated carcinoma, approximately 40 mm in size, with sharply demarcated and raised margins on the Ra (**Lesion C**, Fig. 3). Barium colonography revealed a shadow defect on the lateral image on the right lateral wall of the Ra (Fig. 3). This lesion on the Ra was located on the scar resulting from the previous ESD of the rectal tumor. Contrast-enhanced CT of the thorax and abdomen and PET-CT showed no evidence of metastasis to the lymph nodes or other organs. Therefore, the lesion was surgically resected. The pathological findings of this lesion revealed tumor tissue consisting of moderately large or small irregular ducts infiltrating into the mucosa of the outer membrane. The tumor cells were large and highly columnar, consistent with those observed in moderately differentiated ductal adenocarcinomas. The infiltrated lesion of the carcinoma was poorly differentiated and non-metastatic. There was no metastasis of cancer to the regional lymph nodes; however, venous invasion was observed. Based on these findings, the lesion was diagnosed as a tub2 > por2, pT3 (SS/A), INFb, Ly0, V1b, BD 1, Pn0, pN0, pM0, and pStage IIa (Fig. 3).

We performed cancer multigene panel testing to investigate the correlation between lesions A, B, and C. Formalin-fixed, paraffin-embedded (FFPE) specimens of the three lesions were dissected into 10-µm-thick sections and placed on microscopic slides. DNA extraction was performed using a QIAamp DNA FFPE Tissue kit (Qiagen, Hilden, Germany) according to the manufacturer’s instructions. The quality and quantity of DNA from the three lesions and blood cells were confirmed for next-generation sequence library calculations using a Qubit 1.0 Fluorometer (Life Technologies, Grand Island, NY, USA) and Genomic DNA ScreenTape Analysis (Agilent Technologies, Santa Clara, CA, USA). Subsequently, sequence libraries targeting 468 cancer-related genes from the MSK-IMPACT Clinical Sequencing Cohort (Supplementary Table 1) were prepared from the DNA of the three lesions and blood cells using our sequence library method (see **Supplementary Text**). The resulting pooled libraries were sequenced via paired-end reads using the HiSeq X platform (Illumina, San Diego, CA, USA). Sequencing reads were analyzed and annotated as described in the **Supplementary Text**.

**Fig. 4:**
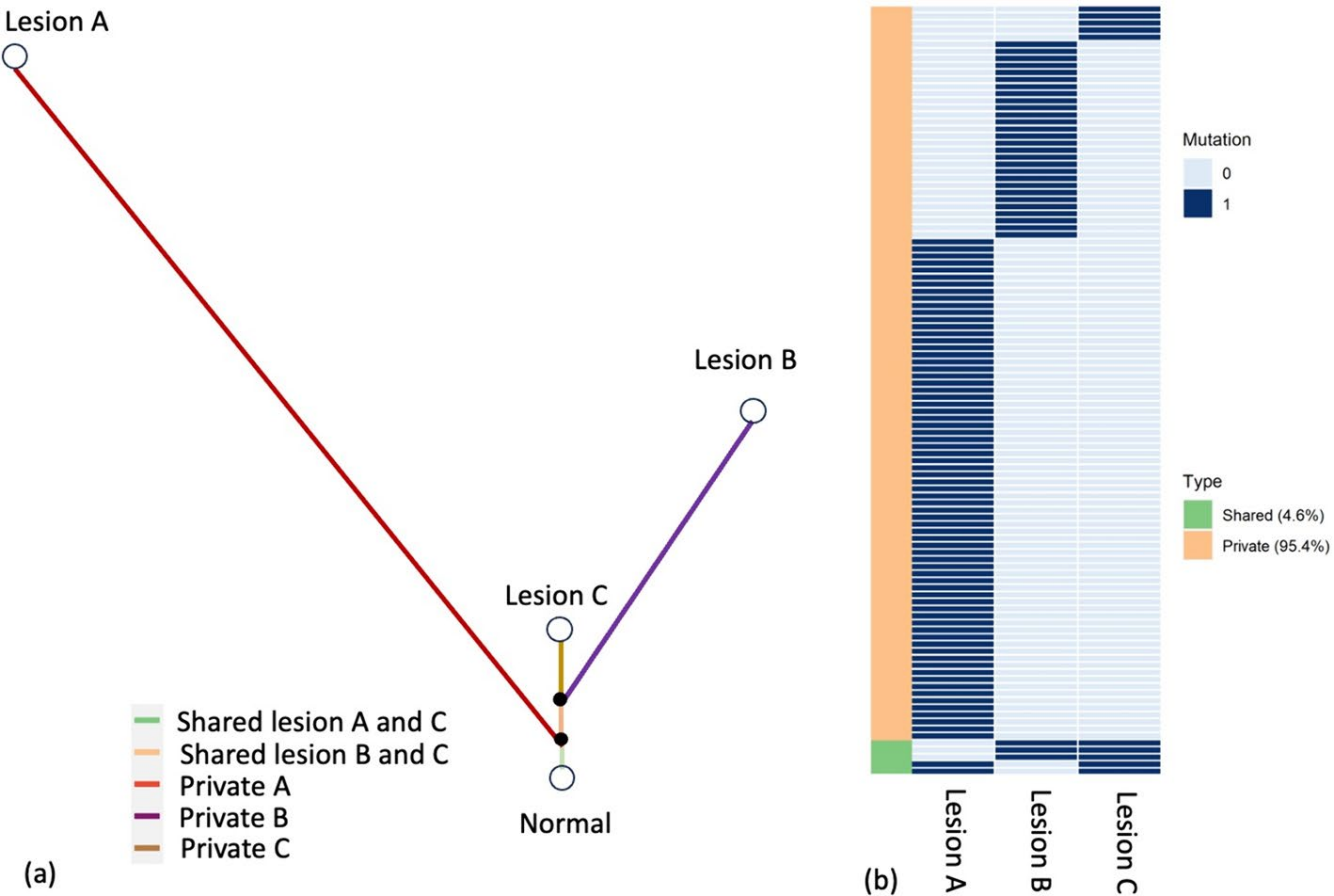
Genomic landscape for lesions A, B, and C. **a** The association of clonal evolution among lesions B and C is shown using a phylogenetic tree. The green bar shows the common variants between A and C (shared lesions A and C). The yellow bar indicates the common variants between B and C (shared lesions B and C). Red, purple, and brown bars indicate variants found only in A, B, and C, respectively. **b** The genomic alterations in lesions A, B, and C are shown as bar graphs. Each line shows each variant in lesions A, B, or C. Shared variants on the right bar indicate common variants between A and C or B and C

Deep sequencing via cancer multigene panel testing revealed that the sequence reads of all samples aligned almost 100% to the reference, with a mean depth of over 300. The numbers of somatic single nucleotide variants (SNVs) in lesions A, B, and C were 73, 31, and 10, respectively. Moreover, the numbers of pathogenic variants in lesions A, B, and C were 8, 5, and 4, respectively. No SNVs were detected in any of the three lesions. However, two SNVs were commonly identified between lesions A and C, and three SNVs were commonly detected between lesions A and C (Fig. 4**, **Supplementary Table 2). The two shared SNVs between lesions A and C were classified as variants of uncertain significance. In contrast, the three shared SNVs between lesions B and C were BRAF, PIK3R1, and TP53, all of which were determined to be pathogenic (Supplementary Table 2).

This study was approved by the Institutional Review Board of Hiroshima University Hospital (approval number: E2012-9990) and conducted in accordance with the principles of the Declaration of Helsinki. Written informed consent was obtained from the patient.

## Discussion

We encountered a case of rectal carcinoma developing on an ESD ulcer resulting from descending colon cancer cell implantation. In this case, three of the four pathogenic mutations detected in the recurrent lesion that occurred on the ulcer following ESD were identical to those observed in the advanced cancer in the descending colon. In contrast, there were no common pathogenic mutations between the colorectal tumor that was treated using ESD and the recurrent lesion. The possibility of hematogenous or lymphatic metastasis as the cause of recurrent lesions cannot be completely ruled out; nevertheless, considering the absence of findings suggestive of lymph node metastasis or metastasis to other sites, along with the patient’s medical history, this result strongly supports the hypothesis that cancer cells derived from colorectal cancer in the descending colon had implanted on the post-ESD ulcer of the rectal tumor, subsequently proliferating and progressing.

Although suspected recurrence of colorectal cancer due to implantation is rare, it is recognized in clinical practice. Both tumor cell implantation and incomplete resection, which result in a residual tumor, are recognized as causes of local recurrence following ESD. Generally, tumor recurrence due to transplantation is suspected when cancer with a submucosal locus of origin is detected at the base of the ulcer of a tumor that had been completely resected via pathological en bloc resection [5]. However, even in cases of negative margins, it is not feasible to pathologically determine the remains of one cell or one glandular duct, and the possibility that the remnant may recur at the base of the ulcer after endoscopic treatment cannot be ruled out. Moreover, as the proliferative activity of recurrent tumors is significantly higher than that of a primary tumor [6], distinguishing between residual and implantation recurrences is difficult. However, there are scattered reports of cancer cells derived from simultaneous multiple colorectal cancers that engrafted on ulcers following endoscopic resection or recurred following endoscopic treatment, as in the present case [7, 8]. It is difficult to confirm that such cases are recurrences of implanted tumors unless the histology of the endoscopically resected tumor differs from that of the implanted tumor cells [7]. However, recent comparisons of genomic information obtained from tumors have indicated that recurrence may be caused by tumor cells from multiple simultaneous colorectal cancers implanting into post-endoscopically treated ulcers [8, 9]. Nishino et al. reported a case in which sigmoid colon cancer was confirmed to have been implanted into an intramucosal rectal cancer site following ESD; KRAS p.G12V mutations were identified in the sigmoid colon cancer as well as recurrent lesions [8]^.^ However, KRAS mutations are common in colorectal tumors, as a result of which the same mutation may be found in all multiple and recurrent tumors. Furthermore, as was the issue in this case, when all multiple and recurrent tumors do not possess KRAS mutations, it is not possible to determine whether implantation or residual recurrence occurred. Therefore, it is important to examine and identify genomic variations in multiple genes when considering whether a patient is presenting with implantation or residual recurrence. Okazawa et al. investigated copy number variants (CNVs) in multiple regions and reported that advanced cancers arising on the post-ESD scars in patients who had undergone ESD for early sigmoid colon cancer or surgery for cecal cancer were residual recurrent lesions [9]. Cancer multigene panel testing, which enables the identification of SNVs and CNVs in multiple cancer-related genes, is a highly reproducible test that is covered by insurance for cancer genome medicine in Japan. We have previously revealed the origin of unknown cancerous and normal tissues via cancer panel testing [10]. Thus, we propose cancer panel testing as a particularly useful tool for distinguishing between implantation recurrence and remnant recurrence, as in this case.

Overall, the currently reported case highlights the importance of endoscopists being aware of the risks of tumor cell implantation when performing endoscopic resection of colorectal tumors in patients with advanced colorectal cancer. Adequate measures should be taken to minimize the risk of tumor cell transplantation when performing endoscopic resection of colorectal tumors in advanced colorectal cancer. During colon resection, saline irrigation is used to wash the lumen to prevent the attachment of exfoliated cancer cells to the anastomosis [2, 11]. Moreover, suturing post-endoscopic ulcers with clips may help prevent cancer cell implantation. This case highlights the significance of post-surgical monitoring, the risk of tumor implantation at previous endoscopic locations, and the potential utility of cancer genomic testing to inform treatment choices and enhance the precision of management strategies for recurrent colorectal neoplasms. The utility of genomic testing should be further investigated using larger scale validation studies across a diverse population of patients.

## Supplementary Information

Below is the link to the electronic supplementary material.

Supplementary file1 (DOCX 14 KB)

Supplementary file2 (PDF 54 KB)

Supplementary file3 (PDF 74 KB)

## Data Availability

The data underlying this article are available in the article and online supplementary material.

## References

[1] Umpleby HC, Fermor B, Symes MO, et al. Viability of exfoliated colorectal carcinoma cells. Br J Surg. 1984;71:659–63. 6478151 10.1002/bjs.1800710902

[2] Hasegawa J, Nishimura J, Yamamoto S, et al. Exfoliated malignant cells at the anastomosis site in colon cancer surgery: the impact of surgical bowel occlusion and intraluminal cleaning. Int J Colorectal Dis. 2011;26:875–80. 21302117 10.1007/s00384-011-1148-1 PMC3117263

[3] Beahrs OH, Phillips JW, Dockerty MB. Implantation of tumor cells as a factor in recurrence of carcinoma of the rectosigmoid. Cancer. 1955;8:831–8. 13240668 10.1002/1097-0142(1955)8:4<831::aid-cncr2820080430>3.0.co;2-t

[4] Oka S, Uraoka T, Tamai N, et al. Standardization of endoscopic resection for colorectal tumors larger than 10 mm in diameter. Dig Endosc. 2017;29(2):40–4. 28425665 10.1111/den.12829

[5] Inoue T, Fujii H, Koyama F, et al. Local recurrence after rectal endoscopic submucosal dissection: a case of tumor cell implantation. Clin J Gastroenterol. 2014;7:36–40. 24523830 10.1007/s12328-013-0445-3 PMC3915078

[6] Tanaka S, Haruma K, Tanimoto T, et al. Ki 67 and transforming growth factor alpha (TGF-α) expression in colorectal recurrent tumors after endoscopic resection. Recent Adv Gastroenterol Carcinogen. 1996;1:1079–83.

[7] Tajika M, Niwa Y, Bhatia V, et al. A first report of tumor cell implantation after EMR in a patient with rectosigmoid cancer. Gastrointest Endosc. 2012;75:1117–8. 21762905 10.1016/j.gie.2011.05.028

[8] Nishino K, Hongo M, Mori N, et al. Implantation of sigmoid colon cancer into the endoscopic resection site of intramucosal rectal cancer: a case report. DEN Open. 2022;3:e193. 36514801 10.1002/deo2.193 PMC9731169

[9] Okazawa Y, Sugimoto K, Ii Y, et al. Local recurrence of submucosal invasive colorectal cancer after endoscopic submucosal dissection revealed by copy number variation. DEN Open. 2023;3:e208. 36742280 10.1002/deo2.208 PMC9889967

[10] Masuda K, Urabe Y, Ito M, et al. Genomic landscape of epithelium with low-grade atypia on gastric cancer after *Helicobacter pylori* eradiation therapy. J Gastroenterol. 2019;54:907–15. 31197475 10.1007/s00535-019-01596-4 PMC6759680

[11] Vogel JD, Felder SI, Bhama AR, et al. The American society of colon and rectal surgeons clinical practice guidelines for the management of colon cancer. Dis Colon Rectum. 2022;65:148–77. 34775402 10.1097/DCR.0000000000002323

