# Supplementary material for: A case of colon cancer implanted on endoscopic resection ulcer certified by cancer genomic testing
Source: Clin J Gastroenterol. 2024 Sep 26;17(6):1047–52. doi: 10.1007/s12328-024-02037-3 (PMC11549191; doi:10.1007/s12328-024-02037-3)
Supplement: Supplementary file 1 — Supplementary file1 (DOCX 14 KB) [file 12328_2024_2037_MOESM1_ESM.docx]

**Supplementary File**

**Sequence library methods**

We developed a sequencing library based on DNA extracted from the tumors and blood cell using the SureSelect XT HS Kit (Agilent Technologies) after fragmenting into 150–200 base pairs using the XT Low Input Enzymatic Fragmentation Kit (Agilent Technologies). The amount of DNA was measured using TapeStation D1000 (Agilent Technologies) before hybridization and used if the prepared library was >500 ng. The SureSelect XT Target Enrichment System (Agilent Technologies) was used to perform target capture. The resulting pooled libraries were sequenced by paired-end reads using the HiSeq X platform (Illumina, San Diego, CA, USA) after quality control check with the High Sensitivity D1000 ScreenTape Assay (Agilent Technologies) (Figure 2).

**Sequencing reads were analyzed and annotated methods**

Sequencing reads were pre-processed using fastp v0.20 and mapped to hg19 using BWA-MEM v0.7.17.23. GATK best practice was used for variant calling. To reduce false positives, somatic mutations were defined as read depths >50 and variant allele frequencies >4%. Copy number analysis was performed using CNVkit v0.9.9 and PureCN v2.0.1.24 Vcf2maf v1.6.21 (https://zenodo.org/record/1185418#.Y_W6cC_3IUs), oncokb-annotator v3.2.1 (https://github.com/oncokb/oncokb-annotator/releases), and InterVar v2.2.225 were used for annotation. We defined alterations as mutations, amplifications, or deletions that are classified as oncogenic or likely oncogenic status in OncoKB (https://www.oncokb.org) or pathogenic or likely pathogenic status in ClinVar (https://www.ncbi.nlm.nih.gov/clinvar/). R package maftools v2.8.5 (https://bioconductor.org/packages/release/bioc/html/maftools.html) was used for data visualization.
