# Supplementary material for: A case of colon cancer implanted on endoscopic resection ulcer certified by cancer genomic testing
Source: Clin J Gastroenterol. 2024 Sep 26;17(6):1047–52. doi: 10.1007/s12328-024-02037-3 (PMC11549191; doi:10.1007/s12328-024-02037-3)
Supplement: Supplementary file 2 — Supplementary file2 (PDF 54 KB) [file 12328_2024_2037_MOESM2_ESM.pdf]

Supplementary 1. Cancer-related genes included in MSK-IMPACT

ABL1	BMPR1A	CRLF2	ERBB4	GATA3	IGF2	MAP3K13	NFKBIA	PLCG2	RB1	SMAD3	TMPRSS2
ACVR1	BRAF	CSDE1	ERCC2	GLI1	IKBKE	MAP3K14	NKX2-1	PLK2	RBM10	SMAD4	TNFAIP3
AGO2	BRCA1	CSF1R	ERCC3	GNA11	IKZF1	MAPK1	NKX3-1	PMAIP1	RECQL	SMARCA4	TNFRSF14
AKT1	BRCA2	CSF3R	ERCC4	GNAQ	IL10	MAPK3	NOTCH1	PMS1	RECQL4	SMARCB1	TOP1
AKT2	BRD4	CTCF	ERCC5	GNAS	IL7R	MAPKAP1	NOTCH2	PMS2	REL	SMARCD1	TP53
AKT3	BRIP1	CTLA4	ERF	GPS2	INHA	MAX	NOTCH3	PNRC1	RET	SMO	TP53BP1
ALK	BTK	CTNNB1	ERG	GREM1	INHBA	MCL1	NOTCH4	POLD1	RFWD2	SMYD3	TP63
ALOX12B	CALR	CUL3	ERRFI1	GRIN2A	INPP4A	MDC1	NPM1	POLE	RHEB	SOCS1	TRAF2
AMER1	CARD11	CXCR4	ESR1	GSK3B	INPP4B	MDM2	NRAS	PPARG	RHOA	SOS1	TRAF7
ANKRD11	CARM1	CYLD	ETV1	H3F3A	INPPL1	MDM4	NSD1	PPM1D	RICTOR	SOX17	TSC1
APC	CASP8	CYSLTR2	ETV6	H3F3B	INSR	MED12	NTHL1	PPP2R1A	RIT1	SOX2	TSC2
AR	CBFB	DAXX	EZH1	H3F3C	IRF4	MEF2B	NTRK1	PPP4R2	RNF43	SOX9	TSHR
ARAF	CBL	DCUN1D1	EZH2	HGF	IRS1	MEN1	NTRK2	PPP6C	ROS1	SPEN	U2AF1
ARID1A	CCND1	DDR2	FAM175A	HIST1H1C	IRS2	MET	NTRK3	PRDM1	RPS6KA4	SPOP	UPF1
ARID1B	CCND2	DICER1	FAM46C	HIST1H2BD	JAK1	MGA	NUF2	PRDM14	RPS6KB2	SPRED1	VEGFA
ARID2	CCND3	DIS3	FAM58A	HIST1H3A	JAK2	MITF	NUP93	PREX2	RPTOR	SRC	VHL
ARID5B	CCNE1	DNAJB1	FANCA	HIST1H3B	JAK3	MLH1	PAK1	PRKAR1A	RRAGC	SRSF2	VTCN1
ASXL1	CD274	DNMT1	FANCC	HIST1H3C	JUN	MPL	PAK7	PRKCI	RRAS	STAG2	WHSC1
ASXL2	CD276	DNMT3A	FAT1	HIST1H3D	KDM5A	MRE11A	PALB2	PRKD1	RRAS2	STAT3	WHSC1L1
ATM	CD79A	DNMT3B	FBXW7	HIST1H3E	KDM5C	MSH2	PARK2	PTCH1	RTEL1	STAT5A	WT1
ATR	CD79B	DOT1L	FGF19	HIST1H3F	KDM6A	MSH3	PARP1	PTEN	RUNX1	STAT5B	WWTR1
ATRX	CDC42	DROSHA	FGF3	HIST1H3G	KDR	MSH6	PAX5	PTP4A1	RXRA	STK11	XIAP
AURKA	CDC73	DUSP4	FGF4	HIST1H3H	KEAP1	MSI1	PBRM1	PTPN11	RYBP	STK19	XPO1
AURKB	CDH1	E2F3	FGFR1	HIST1H3I	KIT	MSI2	PDCD1	PTPRD	SDHA	STK40	XRCC2
AXIN1	CDK12	EED	FGFR2	HIST1H3J	KLF4	MST1	PDCD1LG2	PTPRS	SDHAF2	SUFU	YAP1
AXIN2	CDK4	EGFL7	FGFR3	HIST2H3C	KMT2A	MST1R	PDGFRA	PTPRT	SDHB	SUZ12	YES1
AXL	CDK6	EGFR	FGFR4	HIST2H3D	KMT2B	MTOR	PDGFRB	RAB35	SDHC	SYK	ZFHX3
B2M	CDK8	EIF1AX	FH	HIST3H3	KMT2C	MUTYH	PDPK1	RAC1	SDHD	TAP1	
BABAM1	CDKN1A	EIF4A2	FLCN	HLA-A	KMT2D	MYC	PGR	RAC2	SESN1	TAP2	
BAP1	CDKN1B	EIF4E	FLT1	HLA-B	KNSTRN	MYCL1	PHOX2B	RAD21	SESN2	TBX3	
BARD1	CDKN2A	ELF3	FLT3	HNF1A	KRAS	MYCN	PIK3C2G	RAD50	SESN3	TCEB1	
BBC3	CDKN2B	EP300	FLT4	HOXB13	LATS1	MYD88	PIK3C3	RAD51	SETD2	TCF3	
BCL10	CDKN2C	EPAS1	FOXA1	HRAS	LATS2	MYOD1	PIK3CA	RAD51B	SETD8	TCF7L2	
BCL2	CEBPA	EPCAM	FOXL2	ICOSLG	LMO1	NBN	PIK3CB	RAD51C	SF3B1	TEK	
BCL2L1	CENPA	EPHA3	FOXO1	ID3	LYN	NCOA3	PIK3CD	RAD51D	SH2B3	TERT	
BCL2L11	CHEK1	EPHA5	FOXP1	IDH1	MALT1	NCOR1	PIK3CG	RAD52	SH2D1A	TET1	
BCL6	CHEK2	EPHA7	FUBP1	IDH2	MAP2K1	NEGR1	PIK3R1	RAD54L	SHOC2	TET2	
BCOR	CIC	EPHB1	FYN	IFNGR1	MAP2K2	NF1	PIK3R2	RAF1	SHQ1	TGFBR1	
BIRC3	CREBBP	ERBB2	GATA1	IGF1	MAP2K4	NF2	PIK3R3	RARA	SLX4	TGFBR2	
BLM	CRKL	ERBB3	GATA2	IGF1R	MAP3K1	NFE2L2	PIM1	RASA1	SMAD2	TMEM127	
