# Supplementary material for: A case of colon cancer implanted on endoscopic resection ulcer certified by cancer genomic testing
Source: Clin J Gastroenterol. 2024 Sep 26;17(6):1047–52. doi: 10.1007/s12328-024-02037-3 (PMC11549191; doi:10.1007/s12328-024-02037-3)
Supplement: Supplementary file 3 — Supplementary file3 (PDF 74 KB) [file 12328_2024_2037_MOESM3_ESM.pdf]

Supplementary tale2 detected varints for lesion A, B, and C by cancer genomic testing

LesionA

Start_Position	End_Position	Hugo_Symbol	Chromosome	Variant_Classification	HGVSc	HGVSp_Short	t_depth	t_alt_count	MUTATION_EFFECT
10290899	10290900	DNMT1	chr19	Missense_Mutation	c.456_457delinsTT	p.P153S	342	11	Unknown
10290903	10290903	DNMT1	chr19	Missense_Mutation	c.453A>T	p.R151S	340	11	Unknown
110435098	110435098	IRS2	chr13	Silent	c.3303G>A	p.P1101=	1004	30	Unknown
11170724	11170724	SMARCA4	chr19	Missense_Mutation	c.4772G>A	p.R1591Q	370	14	Unknown
11210263	11210263	MTOR	chr1	Missense_Mutation	c.4490G>A	p.C1497Y	537	142	Unknown
112155042	112155042	APC	chr5	Splice_Site	c.1312+1G>T	p.X438_splice	555	167	Likely Loss-of-function
114711367	114711367	TCF7L2	chr10	Splice_Site	c.381+1G>T	p.X127_splice	470	80	Likely Loss-of-function
116339618	116339632	MET	chr7	In_Frame_Del	c.480_494del	p.C160_Q165d	1392	7	Unknown
116339634	116339675	MET	chr7	In_Frame_Del	c.496_537del	p.I166_A179de	1561	7	Unknown
133759733	133759733	ABL1	chr9	Silent	c.2113C>T	p.L705=	357	15	Unknown
14022038	14022038	ERCC4	chr16	Silent	c.738G>A	p.S246=	800	14	Unknown
140476880	140476880	BRAF	chr7	Missense_Mutation	c.1526G>A	p.R509Q	386	11	Likely Loss-of-function
143033825	143033825	INPP4B	chr4	Missense_Mutation	c.2146G>A	p.V716M	714	22	Unknown
145740446	145740446	RECQL4	chr8	Silent	c.1494G>A	p.T498=	436	10	Unknown
151859842	151859842	KMT2C	chr7	Missense_Mutation	c.10820C>A	p.T3607K	1081	16	Unknown
153251986	153251986	FBXW7	chr4	Frame_Shift_Del	c.1020del	p.V341*	762	23	Likely Loss-of-function
15374309	15374309	BRD4	chr19	Silent	c.1263C>T	p.D421=	772	12	Unknown
156843491	156843491	NTRK1	chr1	Missense_Mutation	c.917A>G	p.D306G	320	8	Unknown
176523670	176523670	FGFR4	chr5	Missense_Mutation	c.2081C>T	p.P694L	856	18	Unknown
17953141	17953141	JAK3	chr19	Missense_Mutation	c.845C>T	p.T282I	376	19	Unknown
181430771	181430771	SOX2	chr3	Missense_Mutation	c.623A>G	p.N208S	959	25	Unknown
187535483	187535566	FAT1	chr4	Splice_Site	c.9076-68_9091del	p.X3026_splice	602	5	Likely Loss-of-function
189526226	189526226	TP63	chr3	Missense_Mutation	c.490G>A	p.A164T	1419	12	Unknown
189526244	189526244	TP63	chr3	Missense_Mutation	c.508G>A	p.D170N	1415	14	Unknown
198260837	198260837	SF3B1	chr2	Missense_Mutation	c.3482T>C	p.M1161T	1049	212	Unknown
212285326	212285326	ERBB4	chr2	Missense_Mutation	c.2975G>A	p.R992H	765	39	Unknown
21562335	21562335	LATS2	chr13	Silent	c.1584G>A	p.S528=	440	11	Unknown
2216560	2216560	DOT1L	chr19	Missense_Mutation	c.2204C>T	p.T735M	578	20	Unknown
2226987	2226987	DOT1L	chr19	Silent	c.4467C>T	p.A1489=	580	15	Unknown
25398284	25398284	KRAS	chr12	Missense_Mutation	c.35G>T	p.G12V	1271	176	Gain-of-function
27105707	27105707	ARID1A	chr1	Missense_Mutation	c.5318T>A	p.L1773Q	798	5	Unknown
30128091	30128091	MAPK3	chr16	Missense_Mutation	c.1038C>G	p.F346L	837	19	Unknown
30713717	30713717	TGFBR2	chr3	Missense_Mutation	c.1117C>T	p.R373C	1028	17	Unknown
31238945	31238945	HLA-C	chr6	Missense_Mutation	c.524G>A	p.R175H	661	43	Unknown
31324710	31324710	HLA-B	chr6	Missense_Mutation	c.98A>T	p.Y33F	342	15	Unknown
31324719	31324719	HLA-B	chr6	Missense_Mutation	c.89G>A	p.R30K	393	15	Unknown
31380568	31380568	DNMT3B	chr20	Missense_Mutation	c.1058C>T	p.T353M	341	8	Unknown
32953616	32953616	BRCA2	chr13	Missense_Mutation	c.8917C>T	p.R2973C	1337	8	Likely Neutral
36940999	36940999	CSF3R	chr1	Nonsense_Mutation	c.340C>T	p.Q114*	877	177	Unknown
3779870	3779870	CREBBP	chr16	Silent	c.5178C>T	p.Y1726=	462	21	Unknown
3781374	3781374	CREBBP	chr16	Missense_Mutation	c.4991G>A	p.R1664H	890	69	Unknown
39742794	39742794	TOP1	chr20	Missense_Mutation	c.1637G>A	p.R546Q	695	21	Unknown
39933150	39933150	BCOR	chrX	Silent	c.1449G>A	p.P483=	455	12	Unknown
41274832	41274832	CTNNB1	chr3	Missense_Mutation	c.1082G>T	p.G361V	495	8	Unknown
41385109	41385109	PTPRT	chr20	Silent	c.852C>T	p.I284=	616	13	Unknown
41747896	41747896	PHOX2B	chr4	Silent	c.873C>T	p.F291=	421	18	Unknown
42845391	42845391	TMPRSS2	chr21	Missense_Mutation	c.871A>G	p.S291G	603	17	Unknown
42852443	42852443	TMPRSS2	chr21	Missense_Mutation	c.643G>A	p.E215K	1090	10	Unknown
4554480	4554480	FGF6	chr12	Missense_Mutation	c.257G>A	p.R86Q	192	5	Unknown
47045068	47045341	RBM10	chrX	Splice_Site	c.2355+42_2431-120del	p.X785_splice	691	22	Likely Loss-of-function
47164983	47164983	SETD2	chr3	Silent	c.1143A>T	p.S381=	664	20	Unknown
48650858	48650858	GATA1	chrX	Missense_Mutation	c.727C>T	p.R243W	223	6	Unknown
49426216	49426216	KMT2D	chr12	Missense_Mutation	c.12272T>A	p.L4091Q	826	19	Unknown
49426611	49426611	KMT2D	chr12	Silent	c.11877G>A	p.Q3959=	1041	15	Unknown
49724798	49724798	MST1	chr3	Missense_Mutation	c.469A>C	p.K157Q	1001	8	Unknown
53226985	53226985	KDM5C	chrX	Missense_Mutation	c.2590C>T	p.P864S	517	16	Unknown
55229284	55229284	EGFR	chr7	Nonsense_Mutation	c.1591C>T	p.R531*	669	7	Unknown
55973990	55973990	KDR	chr4	Silent	c.1326G>A	p.T442=	984	25	Unknown
57861171	57861171	GLI1	chr12	Missense_Mutation	c.968G>A	p.R323Q	723	32	Unknown
63412858	63412858	AMER1	chrX	Silent	c.309C>T	p.A103=	340	18	Unknown
63845614	63845614	ARID5B	chr10	Missense_Mutation	c.1353C>A	p.S451R	1393	10	Unknown
64571819	64571819	MEN1	chr11	Missense_Mutation	c.1835G>A	p.R612H	842	15	Unknown
66765230	66765232	AR	chrX	In_Frame_Del	c.242_244del	p.E81_T82delir	102	3	Unknown
66765234	66765234	AR	chrX	Frame_Shift_Del	c.246del	p.S83Afs*92	106	3	Unknown

66765238	66765239	AR	chrX	Missense_Mutation	c.250_251delinsAG	p.P84S	114	3	Unknown
69588191	69588191	FGF4	chr11	Silent	c.507C>T	p.Y169=	698	22	Unknown
69588843	69588843	FGF4	chr11	Silent	c.393G>A	p.V131=	426	16	Unknown
7172348	7172348	INSR	chr19	Silent	c.1221C>T	p.S407=	693	29	Unknown
7217021	7217021	GPS2	chr17	Nonsense_Mutation	c.500C>G	p.S167*	1584	25	Likely Loss-of-function
81381528	81381528	HGF	chr7	Missense_Mutation	c.533G>A	p.R178Q	753	28	Unknown
89346358	89346358	ANKRD11	chr16	Missense_Mutation	c.6592C>T	p.R2198W	244	20	Unknown
94128970	94128970	EPHA7	chr6	Silent	c.90G>A	p.A30=	939	29	Unknown
9780674	9780674	PIK3CD	chr1	Missense_Mutation	c.1476G>T	p.L492F	488	11	Unknown

LesionB

Start_Position	End_Position	Hugo_Symbol	Chromosome	Variant_Classification	HGVSc	HGVSp_Short	t_depth	t_alt_count	MUTATION_EFFECT
112175305	112175305	APC	chr5	Missense_Mutation	c.4014G>T	p.Q1338H	315	10	Unknown
11905392	11905392	ETV6	chr12	Silent	c.42A>G	p.R14=	303	9	Unknown
128420009	128420009	MAPKAP1	chr9	Missense_Mutation	c.419C>T	p.S140L	568	21	Unknown
128432165	128432165	MAPKAP1	chr9	Missense_Mutation	c.281G>A	p.R94Q	102	4	Unknown
128843230	128843230	SMO	chr7	Missense_Mutation	c.337C>T	p.R113W	192	7	Unknown
131953894	131953894	RAD50	chr5	Missense_Mutation	c.3297T>G	p.D1099E	397	9	Unknown
138403600	138403600	PIK3CB	chr3	Missense_Mutation	c.2182C>G	p.L728V	235	37	Unknown
140453149	140453149	BRAF	chr7	Missense_Mutation	c.1786G>C	p.G596R	484	80	Gain-of-function
142269028	142269028	ATR	chr3	Silent	c.2922G>A	p.T974=	327	11	Unknown
145741444	145741444	RECQL4	chr8	Silent	c.1059C>T	p.Y353=	596	14	Unknown
176715842	176715842	NSD1	chr5	Silent	c.6174C>T	p.Y2058=	262	8	Unknown
18277085	18277085	PIK3R2	chr19	Missense_Mutation	c.1532G>A	p.R511H	204	7	Unknown
18644404	18644404	PIK3C2G	chr12	Missense_Mutation	c.2582T>G	p.I861S	250	8	Unknown
187525565	187525565	FAT1	chr4	Missense_Mutation	c.10514G>A	p.R3505K	332	12	Unknown
18960941	18960941	UPF1	chr19	Silent	c.519C>T	p.D173=	306	8	Unknown
2222404	2222404	DOT1L	chr19	Missense_Mutation	c.3236A>T	p.H1079L	766	24	Unknown
30516930	30516930	PRKD1	chr14	Missense_Mutation	c.19C>A	p.Q7K	72	6	Unknown
36966682	36966682	PAX5	chr9	Missense_Mutation	c.644C>T	p.P215L	301	40	Unknown
40994126	40994126	RAD51	chr15	Splice_Site	c.346+2T>C	p.X116_splice	48	4	Likely Loss-of-function
47028802	47028802	RBM10	chrX	Missense_Mutation	c.106T>C	p.Y36H	218	6	Unknown
5064921	5064921	JAK2	chr9	Silent	c.1095C>T	p.F365=	325	8	Unknown
63412938	63412938	AMER1	chrX	Missense_Mutation	c.229C>T	p.R77W	167	7	Unknown
66941680	66941680	AR	chrX	Missense_Mutation	c.2324G>A	p.R775H	93	4	Unknown
67591111	67591119	PIK3R1	chr5	In_Frame_Del	c.1704_1712del	p.D569_I571de	358	112	Loss-of-function
73994854	73994854	CD276	chr15	Missense_Mutation	c.338G>A	p.R113H	489	13	Unknown
7577120	7577120	TP53	chr17	Missense_Mutation	c.818G>A	p.R273H	241	70	Loss-of-function
78430350	78430350	FUBP1	chr1	Missense_Mutation	c.818G>T	p.G273V	434	10	Unknown
8340356	8340356	PTPRD	chr9	Missense_Mutation	c.5240G>A	p.R1747H	209	6	Unknown
89351871	89351871	ANKRD11	chr16	Missense_Mutation	c.1079C>T	p.P360L	466	74	Unknown
89836623	89836623	FANCA	chr16	Missense_Mutation	c.2267G>A	p.R756H	331	11	Unknown
99482548	99482548	IGF1R	chr15	Missense_Mutation	c.3416G>A	p.R1139Q	302	11	Unknown

LesionC

Start_Position	End_Position	Hugo_Symbol	Chromosome	Variant_Classification	HGVSc	HGVSp_Short	t_depth	t_alt_count	MUTATION_EFFECT
125514405	125514405	CHEK1	chr11	Splice_Site	c.1102-2A>G	p.X368_splice	630	12	Likely Loss-of-function
138665527	138665527	FOXL2	chr3	Missense_Mutation	c.38G>C	p.G13A	121	8	Unknown
140453149	140453149	BRAF	chr7	Missense_Mutation	c.1786G>C	p.G596R	1218	214	Gain-of-function
31324710	31324710	HLA-B	chr6	Missense_Mutation	c.98A>T	p.Y33F	387	13	Unknown
31324719	31324719	HLA-B	chr6	Missense_Mutation	c.89G>A	p.R30K	419	13	Unknown
31504721	31504721	DROSHA	chr5	Nonsense_Mutation	c.1609A>T	p.K537*	716	18	Unknown
48018067	48018067	MSH6	chr2	Missense_Mutation	c.262T>A	p.C88S	226	6	Unknown
67591111	67591119	PIK3R1	chr5	In_Frame_Del	c.1704_1712del	p.D569_I571de	657	259	Loss-of-function
68864659	68864659	PREX2	chr8	Silent	c.30C>T	p.R10=	1285	23	Unknown
7577120	7577120	TP53	chr17	Missense_Mutation	c.818G>A	p.R273H	346	111	Loss-of-function

SNVs writed red character are pathogenic variant, and SNVs writed black character are pathogenic variant.

SNVs located green backgroud are same variant between lesion A and C, and SNVs located yellow backgroud are same variant between lesion B and C.
